# Unveiling the Sex-Based Divide: Exploring Sex Differences in Peripheral Artery Disease

**DOI:** 10.1007/s11883-025-01330-x

**Published:** 2025-09-15

**Authors:** Fouzul Kansul, Alexander B. Crichton, Enikő Pomozi, Trisha L. Roy

**Affiliations:** 1 College of Engineering, Texas A&M University, College Station, TX USA; 2 DeBakey Heart and Vascular Center, Houston Methodist Hospital, Houston, TX USA

**Keywords:** Peripheral artery disease, Sex-based differences, Atherosclerosis, Critical limb-threatening ischemia

## Abstract

**Purpose of Review:**

Peripheral artery disease (PAD) is a leading cause of cardiovascular morbidity and mortality. Despite its growing clinical burden, the disease remains relatively understudied and underdiagnosed relative to other cardiovascular diseases, especially in female patients. While PAD prevalence is similar in males and females, sex-based disparities have been noted in the presentation, diagnosis rates, treatment methods, and clinical outcomes of PAD patients. This article serves to provide an overview of existing knowledge on sex-based differences in the epidemiology, pathophysiology, clinical presentation, management, and patient outcomes of PAD.

**Recent Findings:**

Female patients with PAD remain underdiagnosed and undertreated, with females receiving lower referral rates to supervised exercise therapy and guideline-directed medical therapy despite observing similar benefits from treatment as males. Surgical interventions also see conflicting outcomes in male and female patients, with females now seeing improved outcomes despite prior studies indicating worsened quality of life. Cardiovascular risk factors such as history of smoking, chronic kidney disease, and diabetes also place females at heightened risk of PAD compared to male patients. There are also physiological disparities observed that affect the presentation and diagnosis rates of PAD, with female patients seeing increased platelet reactivity and aggregation yet receiving later diagnoses at more severe stages due to postmenopausal effects. In addition, female patients remain underrecruited in clinical research studies for cardiovascular disease, highlighting a need for further research focused on female patients and sex disparities in PAD.

**Summary:**

This article identifies key disparities between male and female patients with PAD, with inequities noted in the epidemiology, pathophysiology, risk factors, clinical presentation, and treatment approaches and outcomes. Further research is warranted to better understand sex-based differences in PAD and better inform patient treatment decisions.

## Introduction

Peripheral artery disease (PAD) is a progressive and debilitating vascular condition characterized by the narrowing and blockage of lower extremity blood vessels [1, 2]. The spectrum of PAD is broad, with patients being asymptomatic to presenting with intractable rest pain or gangrene, but all are at increased risk of morbidity and mortality [3, 4]. Those with the latter presentation, known as chronic limb-threatening ischemia (CLTI), are at the highest risk, with one year mortality estimated to be 25% [5]. Global estimates indicate that more than 230 million individuals are currently affected with PAD [6, 7]. Prevalence of the disease is predicted to increase by 50% by 2045, primarily due to an ageing population, increase in obesity and diabetes mellitus, and a significant proportion of the population continuing to smoke [7]. Despite its substantial clinical burden, PAD remains relatively under-diagnosed and poorly treated, especially in female patients [2, 8].

While PAD has historically been considered a male-dominant disease, emerging evidence suggests a more equal, if not greater, distribution of disease prevalence in females compared to males [3, 8, 9]. Female PAD patients appear to experience unique pathophysiology, clinical presentations, and treatment outcomes, indicating a need for more personalized treatment approaches. Females with PAD are more likely to be asymptomatic or report atypical symptoms, which can result in delayed diagnosis [3]. Studies also show that female patients face severe functional impairment, poorer quality of life, and worse post-procedural complications following surgical intervention compared to males [4, 6, 9, 10]

**Fig. 1:**
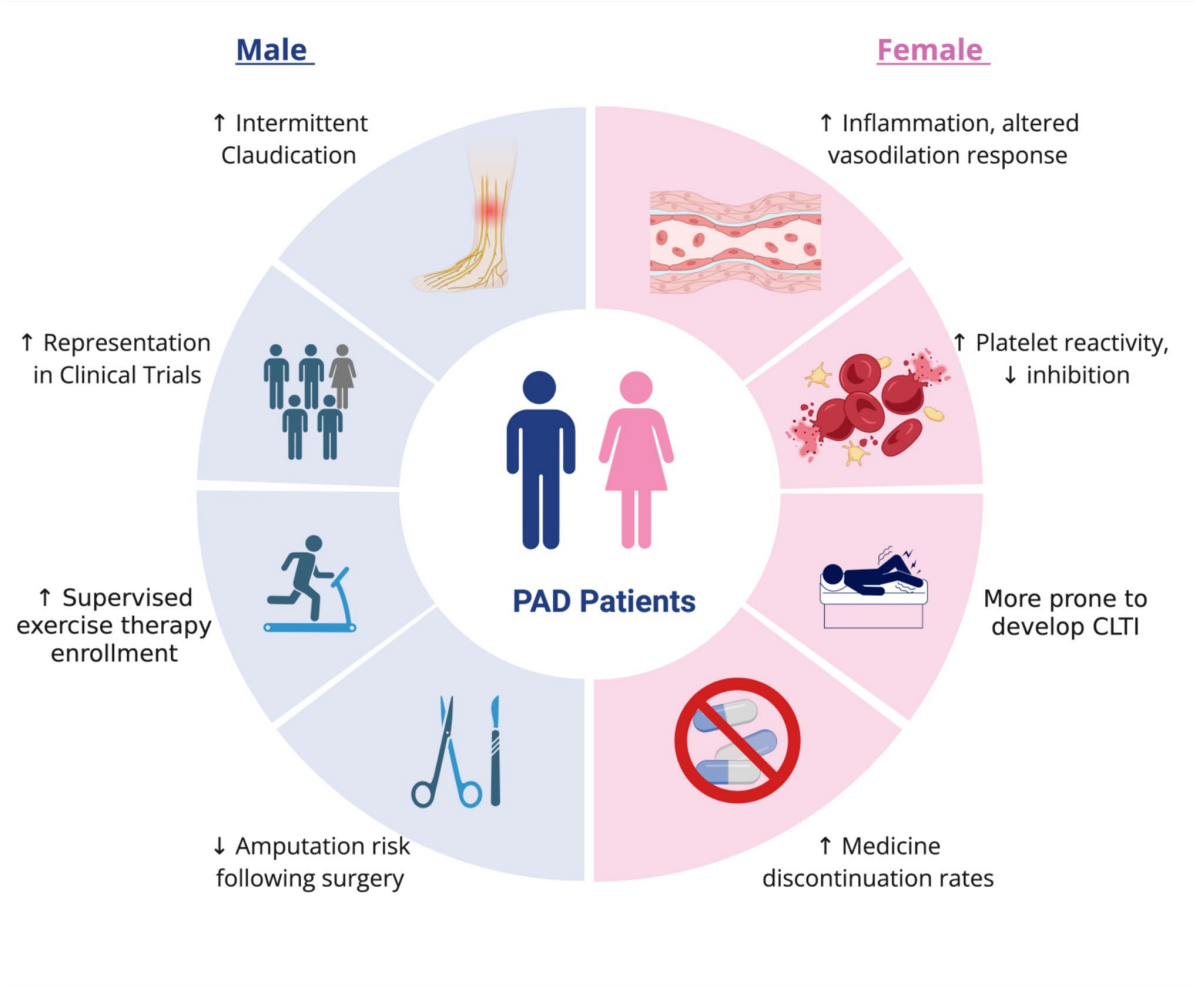
Overview of sex-based differences in PAD patients. Created in BioRender. Kansul [28] https://BioRender.com/2jlf5ou

Despite these disparities, current research on PAD remains limited in its focus on sex-based differences, with clinical guidelines still reflecting data obtained from male-majority cohorts [8, 9, 11]. This review aims to explore the sex-based differences in PAD, comparing variations in epidemiology, pathophysiology, risk factors, clinical presentation, treatment approaches, and patient outcomes to better understand disease progression in male and female patients and inform treatment decisions (Fig. 1).

### Epidemiology and Underrepresentation in Landmark Trials

PAD, which affects 7–12 million adults in the USA, has historically been associated as a disease that predominantly affects the male population [3]. Describing the true epidemiology of PAD is challenging given the different diagnostic approaches that can be used; however, it is likely that the disease prevalence in female patients is underreported [12]. The easiest screening test for PAD is an ankle-brachial index (ABI) given its non-invasive nature and when used as a diagnostic tool, the prevalence of PAD is similar in both males and females [12–14]. However, ABI is unreliable in many diabetic patients, who represent the most rapidly growing population with PAD, as they often have incompressible vessels that falsely elevate ABI readings, potentially leading to underdiagnosis [15]. Studies have suggested that there are both ethnic and sex differences in ABIs, and there are suggestions that sex-specific ABI guidelines could be beneficial in the diagnostics of PAD in female patients [16, 17].

Further challenges in estimates occur due to the atypical presentation of females with PAD. Symptomatology of cardiovascular diseases have been based on the classical presentation of males, which includes chest pain, midback pain, nausea, dyspnea, and fatigue [18]. However, it is known that female patients often do not present in the same way which can lead to delayed diagnosis [19]. In PAD, many females are asymptomatic, have atypical exercise induced symptoms or have overlapping symptoms due to musculoskeletal diseases which can shroud the true diagnosis of PAD [20]. This ultimately results in the underdiagnosis of PAD, which is significant, as even in patients who are completely asymptomatic, there remains a significant risk of cardiovascular morbidity and mortality.

At the most severe end of the spectrum of PAD is CLTI, in which patients present with intractable rest pain, non-healing ulceration in the presence of PAD or gangrene which again is traditionally expected to affect significantly more males than females [21]. However, an analysis of patients presenting with CLTI to hospitals suggested that the gap is much less, with a study representing almost 200,000 admissions showing that 43% of patients were female [22]. Despite this, however, female patients are frequently under-represented in trials. In the BASIL-2, BASIL-3 and BEST CLI trials which are the largest CLTI randomized clinical treatment trials in the last decade, female inclusion rates were between 19–35% [21, 23, 24]. The implications of this underrepresentation could be significant, as sex-based outcomes following different treatment strategies are well evidenced in the literature and are discussed further in this article.

### Pathophysiology

The pathophysiology of PAD is comprised of various hormonal and biological factors, including endothelial dysfunction, oxidative stress, thrombosis, platelet activation, sex hormone levels, and vascular remodeling. Biological sex plays an important role in determining the extent and presentation of these factors in PAD patients.

Research suggests that male sex hormones, particularly testosterone, have a protective effect against PAD in males, a benefit not widely observed in female patients [2]. A cross-sectional study of 2703 older male subjects revealed that signs of intermittent claudication, a common symptom of PAD in males, was linked to lower testosterone or dihydrotestosterone levels [25]. An inverse relationship between serum androgen levels and PAD symptoms in older males was also demonstrated, with male PAD patients demonstrating lower testosterone and sex hormone binding globulin concentrations [2]. Testosterone deficiency has also been linked to increased oxidative stress, endothelial dysfunction, and inflammation, all of which contribute to increased atherosclerosis [26]. Literature suggests that testosterone may play an independent role in atherosclerosis development and PAD pathogenesis for elderly female patients as well [26]. The findings of existing studies imply that reduced androgen exposure may cause the onset and development of symptomatic PAD [2].

Estrogen, on the other hand, has been shown to protect against PAD in female patients through its regulation of vascular functions and proangiogenic qualities [2, 3, 26]. While clinical and epidemiological studies do not demonstrate strong associations between serum estrogen levels and PAD development, animal studies reveal that estrogen signaling may promote neovascularization and assist in recovery from ischemic PAD. The proangiogenic effects of estrogen are also seen in male animal models, where treatment with estrogen showed improved blood flow [2, 3]. Further research is needed to identify the mechanisms through which estrogens and androgens protect individuals from PAD.

Sex-based differences in endothelial dysfunction play a significant role in PAD pathophysiology as well. Endothelial dysfunction in PAD is linked to oxidative stress, arterial stenosis, and inflammatory activation in skeletal muscle cells [27]. Female PAD patients display greater inflammation, reduced angiogenesis, and altered vasodilation responses compared to males [6]. A study evaluating sex-based differences in vessel wall morphology in 34 end-stage patients (16 females) demonstrated that, while plaque morphology appears to converge between sexes, there is an increased prevalence of luminal thrombus in post-menopausal female patients [28]. Females also demonstrate increased platelet reactivity and greater platelet aggregation, which can affect the development and progression of PAD [28, 29]. Majumdar et al. has also shown differences in platelet function between male and female patients with PAD. Despite having lower rates of significant cardiovascular diseases and risk factors, females were found to have significantly higher platelets reactivity and aggregation and lower levels of platelet inhibition [30]. These differences highlight the sex-based disparities in PAD at the biological and physiological levels and demonstrate a need for more tailored treatment approaches.

### Risk Factors

PAD is typically evaluated by the presence of traditional cardiovascular risk factors, including smoking, diabetes, hypertension, and chronic kidney disease [4, 31]. While these risks are seen in male and female patients alike, sex-based differences are observed in the prevalence and impact of certain factors on disease presentation and patient quality of life.

Smoking is a significant yet preventable risk factor that contributes to PAD. Although the burden of smoking has decreased in the past few decades due to public awareness efforts, approximately one quarter of all PAD-related deaths remain linked to tobacco use [32, 33]. Studies demonstrate that females with a history of smoking are at greater risk of PAD compared to males, with the risk of cardiovascular disease being 25% greater in female smokers than in male smokers when relatively compared with non-smokers of the same sex [33, 34]. Diabetes is the second most significant risk factor for PAD following smoking, with female patients displaying a greater sex-specific risk for atherosclerotic disease compared to males [35]. Meta-analyses suggest that this increased risk could be attributed to biological variances between males and females, such as in adipose tissue distribution, hormone influences, or genetic factors [36].

Hypertension, a leading risk factor for PAD, is typically considered more predominant in males [5]. However, emerging studies showcase varying incidences of hypertension between sexes, suggesting that blood pressure is a sexually dimorphic trait that varies in females and males throughout their lifetimes [37, 38]. Females also demonstrate a significant increase in blood pressure during menopause, indicating that age and hormonal fluctuations may contribute to their risks of PAD [31]. Chronic kidney disease (CKD) is another high-risk factor for PAD. Newer studies of sex-based disparities in vascular disease development in CKD cohorts indicate that females with CKD have a higher PAD risk compared with males at younger ages, with the risks equalizing between sexes at ages > 70 years [39]. As CKD involves other cardiovascular risk factors such as hypertension and diabetes, it is possible that the overlap of risk factors is what increases PAD risk in younger female patients.

Hypercholesterolemia is also a significant risk factor for PAD [40]. Over 40% of female patients had a total cholesterol of > 200 mg/dL in the NHANES data and the proportion of females with high total cholesterol levels is higher in females > 50 years old compared to males in the same age range [41]. Small increases in low density lipoprotein levels in females are also significantly associated with heightened risks of morbidity and mortality, highlighting the need for tight control of cholesterol [42]. Female patients are also at increased risk of autoimmune diseases and anxiety and depression, both of which are associated with an increased risk of PAD [43, 44]. Overall, sex-based differences in PAD risk factors indicate that females with multiple risk factors are at higher risk of developing PAD [4].

### Clinical Presentation

The clinical presentation of PAD is typically defined by symptoms such as intermittent claudication, rest pain, and non-healing wounds/gangrene. However, the majority of PAD patients are asymptomatic or present with atypical symptoms [9, 45]. This is especially pronounced in female patients, leading to delayed diagnosis and treatment for PAD compared to male patients. A systematic review of multiple studies involving a total of 1,929,966 PAD patients confirmed that females were less likely than males to present with intermittent claudication [46]. Instead, female patients with PAD are more prone to develop critical limb ischemia, with some studies suggesting a 2-fold prevalence compared to male PAD patients [7, 47].

Existing risk factors and comorbidities, such as chronic kidney disease and diabetes, may also uniquely impact the presentation of PAD patients based on their sex. A multi-center study of 3,174 participants with CKD revealed that females demonstrated a 1.53-fold higher risk of PAD compared to males, with significant variation by age [39]. Younger females showed an abnormally increased risk of developing PAD, which normalized after the age of 70, while older males had significantly higher PAD risks compared to younger males[39]. While this data conflicts with multiple existing studies reporting PAD as more prevalent in elderly females, this study suggests that comorbidities, such as chronic kidney disease, can affect the clinical presentation of PAD disproportionately by sex [8, 39].

It also appears that PAD when symptomatic affects females more significantly. Studies show that female PAD patients have less muscle strength, are slower when walking, and can only walk shorter distances than male patients, suggesting that differing supportive treatment needs for supervised exercise therapy are required between the sexes [20, 48]. Earlier and more specific screening strategies for PAD in female patients with significant comorbidities are warranted given the worse outcomes, early onset, and rapid functional decline [7].

### Treatment Approaches and Patient Outcomes

Treatment methods for PAD primarily aim to limit progression of the disease while improving quality of life for patients. These treatments include exercise therapy, pharmaceutical therapy, and surgical or endovascular interventions. While the common goal of these approaches is to minimize risk factors and lower the incidence of adverse cardiovascular and limb complications, significant sex-based disparities in applied PAD treatments and outcomes remain.

Exercise is considered a first line of therapy for PAD, as regular and supervised exercise therapy (SET) can improve muscle performance, reduce pain, and counter intermittent claudication in PAD patients [27]. Additionally, exercise induces anti-oxidative effects, modulating antioxidant enzymes and regulating endothelial genes associated with improving vascular health. Despite the proven benefits of SET, utilization of this treatment method remains low for PAD patients – even so, males are more likely to be referred for such therapy compared to female patients [49]. A study evaluating SET in individuals with intermittent claudication revealed that both male and female patients saw improvements in absolute claudication distance and functional walking distance after therapy [50]. However, female patients demonstrated a significantly lower increase in absolute claudication distance during the first three months and shorter absolute walking distances after 1 year compared to male patients in the same cohort, yet adherence to SET programs are similar between males and females [50, 51]. While SET remains independently effective for both sexes, female patients may require a more intensive or tailored exercise protocol to achieve recovery rates similar to male patients.

Pharmaceutical therapy is paramount in the initial treatment for PAD patients and includes prescription of statins and antiplatelets. Risk factor management with antihypertensives, antihyperglycemics, and supportive measures in smoking cessation are also crucial. While these guideline-directed medical therapies are shown to reduce risk in PAD patients, prior evaluations have shown differences in treatment outcomes on the basis of sex [52]. An analysis of 9810 PAD patients at the University of Colorado revealed that females were less likely than males to receive guideline-directed medical therapies, regardless of concurrent diseases, symptomatic PAD, or prior lower extremity vascularization treatment [52]. The benefits of statins are well evidenced in both males and females for reducing cardiovascular disease, yet female patients are less likely to be prescribed statins, are more likely to discontinue them, and are less likely to be on a high intensity dosage [53]. Overall, adherence to medication is also notably lower in female patients, which further accelerates PAD progression and functional decline [11, 54]. Cilostazol, a key antiplatelet treatment used to improve intermittent claudication, has shown benefits for both males and females with PAD, contributing to improved initial and absolute walking distances [55–57]. A pooled analysis of nine randomized control trials assessing the use of cilostazol for intermittent claudication reported no key differences in treatment outcomes between male and female patients. Another study assessing the effects of rivaroxaban, an anticoagulant, in PAD patients showed that both male and female patients benefited from the treatment, with reduced risk of cardiovascular and limb events. However, females displayed a higher discontinuation rate, impacting the observed benefits [54]. The low usage and adherence of guideline-directed medical therapy in females with PAD could potentially contribute to the worse clinical outcomes they face, such as high risk of cardiovascular events, limb amputations, and unplanned readmissions despite similar disease severity to males [11, 49, 52].

Surgical and endovascular interventions are typically recommended for patients with more severe PAD. Females and males have a similar rate of endovascular treatments for PAD, with treatment type linked to disease severity rather than patient sex [8]. On the other hand, surgical intervention is more prevalent for male patients, with existing literature suggesting that female patients appear to have worse outcomes following surgical procedures [58]. Female patients are placed at higher risk of graft failure, wound infections, and limb loss following surgical revascularization [8, 28, 30, 49]. A study evaluating 843 patients (41.2% female) following lower extremity bypass surgery reported higher major amputation rates after one year in females compared to males, despite similarities in racial and ethnic distribution, comorbidities, and surgical indications [59]. Another observation from 2002–2011 revealed that females with PAD were more likely than males to undergo transfemoral amputation, with the disparity consistent throughout the study timeframe [49]. However, a more recent analysis of the BEST-CLI trial suggests that females see significantly better outcomes following surgical intervention than endovascular therapy when evaluated within an all-female cohort [60]. Females are also shown to have improved amputation-free survival rates at 1 year compared to male PAD patients, suggesting that additional research is needed to evaluate sex-based differences in revascularization outcomes [60].

Disparities are apparent following endovascular interventions as well. A retrospective analysis of 247,981 patients receiving index revascularization procedures for PAD revealed that females were significantly older than males at the time of treatment selection and displayed higher overall mortality and increased gangrene, yet lower amputation rates compared to males [61]. Another study of 3434 PAD patients receiving endovascular therapy from the K-VIS ELLA registry revealed that females have higher rates of death, myocardial infarction, major amputation, complex lesions, and procedural complications [62]. Studies have also shown that female patients are less likely to undergo stenting in the femoropopliteal arteries and were more likely to be treated with angioplasty alone whereas there was no difference in treatment choice in the iliac arteries [63]. Sex differences are also apparent in antiplatelet therapy performance in post-intervention patients. In a study of 181 PAD subjects (32% female) receiving antiplatelet and anticoagulant medications following intervention, female patients demonstrated greater clot strength, higher platelet reactivity, and reduced platelet inhibition compared to males on the same medication regimen. This significant difference places female PAD patients at increased risk of increased thrombotic events following revascularization procedures [29].

Given the unfavorable outcomes female patients face post-procedure, care should be taken to evaluate the ideal intervention method for PAD patients. A promising study conducted at Houston Methodist showcased the potential of MRI, specifically ultrashort echo time (UTE) sequencing, to characterize plaque components before intervention. This approach could be used to evaluate lesion morphology and predict the success rates of different therapies, providing a more tailored treatment approach and potentially improving patient outcomes [64]. Other emerging tools to evaluate treatment efficacy are platelet mapping and thromboelastography, which provide a comprehensive overview of blood samples to determine the likelihood of clotting [29]. These approaches can improve assessment of coagulation in patients post-procedure, allowing for early intervention if necessary (Table 1).

**Table 1:** Sex differences in pad epidemiology, pathophysiology, risk factors, clinical representation, and treatment approaches/outcomes

Sex-based differences in PAD	
Epidemiology	Disease prevalence of PAD in female patients is underreported [12]
	There are lower rates of female enrollment in cardiovascular randomized clinical trials [22, 24]
	Intractable rest pain, non-healing ulceration, and gangrene are more common in male than female patients [21]
Pathophysiology	Protective effect of estrogen against PAD observed in female patients [2, 3]
	Female patients show higher platelet reactivity/aggregation and lower platelet inhibition [30]
	There is greater inflammation, reduced angiogenesis, and altered vasodilation in females compared to males [6]
Risk Factors	25% higher PAD risk in female vs male smokers [33, 34]
	Increased PAD risk in female diabetic patients [35]
	Postmenopausal BP spike increases PAD risk in female patients [31]
	Female CKD patients < 70 yrs have higher PAD risk than males [39]
	Increased cholesterol in females vs males > 50 yrs [41]
	Females at greater risk of autoimmune disease, anxiety, and depression which are associated with PAD [43, 44]
Clinical Presentation	Delayed diagnosis and treatment for PAD in female vs male patients [19]
	Females more likely to be asymptomatic or have atypical exercise induced symptoms [20]
	Presence of risk factors such as CKD disproportionately impacts PAD presentation by sex [8, 39]
	Males more likely to present with intermittent claudication than females [46]
Treatments/Outcomes	Female PAD patients less likely to be referred to SET compared to males [49]
	Cilostazol, statins and Rivaroxaban benefit both sexes, but females have greater discontinuation rates [53, 54]
	Improved amputation-free survival rates in female vs male PAD patients following bypass as shown in the BEST CLI trial [60]

### Future Directions

PAD is complex in its development, diagnosis and management, and to some degree this intricate matrix could account for some of the differences between male and female patients described in this article. However, there are also significant and unexplained differences in outcomes.

Female patients represent a significant proportion of people with PAD, and this is replicated in hospitalized patients with CLTI. Despite this, landmark randomized trials that we use to guide our treatment for these patients consistently underrecruit female participants [21, 23, 24]. It is vitally important that trials are representative of our population so that appropriate decisions for clinical management can be made. Alternatively, studies that are adequately powered to evaluate differences in outcomes based on sex would provide adequate evidence for informed decision making. This finding also correlates with the literature on presentations of PAD, which often fail to highlight the need for greater awareness of concomitant diseases that can mask PAD. The downstream effect of this is underdiagnosis and therefore undertreatment. Studies show that female patients are undertreated, notably in the pharmaceutical management of PAD and lower referral rates to SET, despite equal adherence to supervised program treatment [49–51, 53]. Adherence to medications is also low though, and it would be of merit to this significant population of patients affected by PAD to investigate this further.

The choices of interventional treatment between males and females will owe partly to differences in decision making and presentation, and there is a need for identifying why these differences occur. A key component of this though, will be the pathological differences in disease morphology. Traditionally, this would have been most easily assessed with pathological or physiological assessments as described in the currently available literature that has been discussed in this article [28, 30]. Imaging advances, however, have taken significant strides and provide an ability to evaluate the arteries in their entirety rather than sections. It also allows a wider evaluation of patients who are undergoing treatment, rather than those only undergoing amputation or those who have deceased. UTE non-contrast MRI imaging has the ability to differentiate vessel wall plaque characteristics as shown by Roy et al. [65]. This technique would not only identify differences in plaque morphology at first presentation but could also be used to investigate vessel wall response differences between males and females, as well as the differences in response to the wide variety of available treatments.

## Conclusion

Biologic sex significantly affects the overall outcomes of patients with PAD, but the reasoning for this has only been partially explained with currently available evidence. From pathophysiology through to choices of intervention, lies a complex interaction of multiple variables. Basic science, translational and clinical trial research should incorporate key sex-based outcomes of interest into their study design to identify and resolve health inequities.

## Key References

- Farber A, Menard MT, Conte MS, Kaufman JA, Powell RJ, Choudhry NK, et al. Surgery or Endovascular Therapy for Chronic Limb-Threatening Ischemia. New England Journal of Medicine. 2022;387(25):2305–16.
  - Randomized clinical trial to evaluate the Best Endovascular vs Best Surgical Therapy in Patients with CLTI (BEST-CLI).
- Bradbury AW, Moakes CA, Popplewell M, Meecham L, Bate GR, Kelly L, et al. A vein bypass first versus a best endovascular treatment first revascularisation strategy for patients with chronic limb threatening ischaemia who required an infra-popliteal, with or without an additional more proximal infra-inguinal revascularisation procedure to restore limb perfusion (BASIL-2): an open-label, randomised, multicentre, phase 3 trial. Lancet. 2023;401(10390):1798–809.
  - Comparison study of vein bypass first vs best endovascular treatment first approaches that emphasized need for personalized treatment decisions.
- Bradbury AW, Hall JA, Popplewell MA, Meecham L, Bate GR, Kelly L, et al. Plain versus drug balloon and stenting in severe ischaemia of the leg (BASIL-3): open label, three arm, randomised, multicentre, phase 3 trial. BMJ. 2025;388:e080881.
  - Randomized multicenter study that showed no significant differences in clinical outcomes between plain vs drug balloon and stenting in patients with severe limb ischemia.
- Pabon M, Cheng S, Altin SE, Sethi SS, Nelson MD, Moreau KL, et al. Sex Differences in Peripheral Artery Disease. Circ Res. 2022;130(4):496–511.
  - Comprehensive review article examining sex disparities in the epidemiology, diagnosis, treatment, and outcomes of PAD.

## Data Availability

No datasets were generated or analysed during the current study.
